# Affective Decision-Making and Tactical Behavior of Under-15 Soccer Players

**DOI:** 10.1371/journal.pone.0101231

**Published:** 2014-06-30

**Authors:** Adeilton dos Santos Gonzaga, Maicon Rodrigues Albuquerque, Leandro Fernandes Malloy-Diniz, Pablo Juan Greco, Israel Teoldo da Costa

**Affiliations:** 1 Physical Education Departament - Universidade Federal de Viçosa, Viçosa, Minas Gerais, Brazil; 2 Mental Health Department, Universidade Federal de Minas Gerais, Belo Horizonte, Minas Gerais, Brazil; 3 Sports Departament - Universidade Federal de Minas Gerais, Belo Horizonte, Minas Gerais, Brazil; Centre national de la recherche scientifique, France

## Abstract

Affective decision-making is a type of Executive Function related to cost benefit analysis in situations where gains and losses imply direct consequences for the subject. The purpose of this study was to explore the influence of the affective decision-making on tactical behavior in soccer players under the age of 15 years old. The System of Tactical Assessment in Soccer (FUT-SAT) was used to assess tactical behavior. To evaluate affective decision-making, we used the neuropsychological test called The Iowa Gambling Task (IGT). The values of the offensive, defensive and game tactical behavior of participants were used to create performance groups. The low (≤25%) and high (≥75%) groups, according to offensive, defensive and game tactical behavior, were compared and shown to be different. The values of the IGT net score of the participants with low and high tactical behavior were compared using the non-parametric Mann-Whitney test. Statistically significant differences between the groups were observed for Defensive Tactical Behavior (Z = −3.133; p = 0.002; r = −0.355) and Game Tactical Behavior (Z = −2.267; p = 0.023; r = −0.260). According to these results, it is possible to state that affective decision-making can influence the tactical behavior of under-15 soccer players.

## Introduction

For many years, physical features have received the most attention as the main factors for players achieving high levels of performance in soccer [1], [2], [3]. However, in recent years, some concerns have been allocated to the development of tactical skills as an important feature of successful performance in soccer players and teams [4], [5]. These concerns are justified by the dynamic and complex features of the game, which is characterized by a cooperation-opposition relationship between teammates and opponents [6].

Playing well requires repeatedly performing tactical skills efficiently throughout the match [7]. During a soccer game, players are requested to coordinate their actions to recover, retain and move the ball to attack as well as to create goal scoring situations, which requires well-developed tactical skills to achieve successful performance [5]. According to Gréhaigne and Godbout [5], tactical skills refer to the ability of a player to make and execute an appropriate decision in any given situation according to game constraints.

To perform successfully, players should present well-developed tactical knowledge, which has been categorized as declarative (“what to do”) and procedural (“doing it”) [8]. Studies have shown that players with a higher level of performance present better tactical knowledge in comparison to those players with lower levels of performance [9], [10]. In general, players with a better understanding of the game are more able to perform successful tactical behavior and to make correct tactical decisions in game events that enable them to achieve a high level of performance [11].

Due to the constant changes in game environment, players are also forced to inhibit pre-planned responses, anticipate actions and coordinate body segments based on the complex and dynamic flow of sensory information [12]. Thus, to perform efficient tactical behaviors and achieve high performance levels, the players need to present well-developed cognitive abilities [13].

The neuropsychology field has used the term executive function (EF) as an “umbrella” construct comprising a wide range of cognitive processes and behavioral competencies to describe actions that involve inhibiting responses, decision-making, effortful and flexible organization of actions, anticipatory actions, and strategic planning [14]. Although EF is often considered a domain-general cognitive function, researchers have described a distinction between metacognitive (associated with circuitry involving the dorsolateral prefrontal cortex) and emotion/motivation related (associated with the orbitofrontal cortex) EF [15].

Affective decision-making is a decision process with emotional consequences marked by meaningful rewards and/or losses [16]. Even this process demands more purely cognitive skills, such as attention and working memory, which are necessary to keep track of the consequences of previous choices, though making affective decisions relies mainly on EF, as it is more related to emotional and motivational processes [17], [18]. Across the lifespan, making decisions that will bring greater long-term gains instead of immediate rewards is a crucial skill that is developed during childhood and adolescence [19], [20].

Vestberg and colleagues [21] explored the influence of EF in predicting the success of soccer players. The authors verify that “high division players” had better performance than “low division players” on some EF measures. In addition, the authors argued that in a selection process of future soccer players, decisions should include not only judgments of physical capacity, ball control and how well the player performs but also need to include measures of executive functioning using validated neuropsychological tests. Thus, the authors concluded the paper with “… the present study may change the way ball-sports are viewed and analyzed and how new talents are recruited.” (Vestberg et al., 2012, p.4). However, the cognitive abilities tested by these authors in their study were more cognitive-type EF.

As observed for this type of EF, those processes related to emotional/motivational components of EF, such as affective decision-making, seem to be important to performance in soccer. Because a player’s decisions are related not only to contextual, perceptual and cognitive aspects of the game but also involve motivational and emotional factors, it is important to assess the relationship between affective decision-making and performance in soccer players [17]. Thus, the purpose of this study was to explore the influence of affective decision-making on tactical behavior in young soccer players.

## Methods

### Participants

This study comprised 9,713 tactical behaviors (4,698 offensives and 5,015 defensives) performed by 153 under-15 (U-15) soccer players (Mean age = 14.35; SD = 0.63). All participants were engaged in regular training sessions in soccer at least three times a week. Moreover, they were participating in a regional level championship for their age category.

Before the data collection, the directors of teams signed a document authorizing the research. Additionally, the participants and their parents signed a legal consent allowing data collection and the use of the data for research purposes. This study was authorized by the Ethics Committee (Of. 132/2012/CEPH/01-12-11) of the Universidade Federal de Viçosa.

### Task

#### Tactical Behavior

To evaluate the tactical behavior of the players, the System of Tactical Assessment in Soccer (FUT-SAT) was used [11], [22]. The conceptual structure of FUT-SAT is based on the ten core tactical principles of soccer, being five for the offensive phase: penetration, offensive coverage, depth mobility, width and length and offensive unity; and five for the defensive phase: delay, defensive coverage, balance, concentration and defensive unity [22]. These principles were chosen since they represent the core aspects of the process of teaching and training of tactical skills. Besides that, this set of principles objectively measures players’ motion according to the management of playing space performed by them.

FUT-SAT comprises two macro-categories, seven categories and 76 variables that are organized according to the type of information dealt with by the system (for more details see [22]). The Macro-Category Observation comprises three categories and 24 variables. This Macro-Category, named Tactical Principles, comprises ten variables. The category Place of Action in the Game Field features four variables and the category Action Outcomes features ten variables.

The Macro-Category Outcome features four categories and 52 variables. In this Macro-Category, all four categories Tactical Performance Index (TPI), Tactical Actions, Error Percentage and Place of Action Related to the Principles (PARP) comprise the same thirteen variables. The Macro-Category Outcome is so called becasue its variables are dependent on the information provided by the variables that compose the Macro-Category Observation.

The FUT-SAT’s field test (Goalkeeper +3 vs. Goalkeeper +3) is performed during four minutes in an area of 36 meters long by 27 meters wide, according to the official laws of soccer, except by the offside rule.

To assess tactical behavior we used players’ Offensive, Defensive, and Game Tactical Performance Index values.

#### Affective Decision-making Task

The Iowa Gambling Task (IGT) is an experimental neuropsychological task designed to study the integration of emotion and cognition in decision-making processes. It simulates real-life decision-making with uncertainty concerning premises and outcome as well as reward and punishment. Impaired performance has been found in patients with bilateral damage to the ventromedial prefrontal cortices [23].

A computerized Brazilian version of the IGT was used [24]. Starting with a $2,000 loan of fake money and with the instructions to win as much money as possible, the subjects were told to choose one card at a time from one of four decks (A, B, C, D). Immediately after every choice, the subjects received a financial reward, although in some cases they also received a financial punishment. Two of the decks (A, B) were disadvantageous and resulted in immediate large rewards, but also in higher punishment at unpredictable points. The other two decks (C, D) were advantageous and resulted in immediate modest rewards, but lower rate of punishment as well. In the long run, choosing from the advantageous decks would result in a net gain, while choosing from the disadvantageous decks would result in a net loss. The subjects were informed that some (but not which) decks were more advantageous and were warned to keep away from the disadvantageous decks. The score on the IGT was defined as the number of choices from the advantageous decks minus the number of choices from the disadvantageous decks over 100 attempts.

### Procedures

The first test performed by participants was FUT-SAT. This test was performed according to the published protocol [11], [22]. In the next phase, the participants came to a room individually to perform the IGT neuropsychological test. For this test, they were invited to sit in a comfortable chair in front of a computer. In addition to being given instructions on the computer screen, the participants were read to by the instructor, who also ensured the computational skills of participants for performing the task. The test started after the participants affirmed their understanding of the task. The test ended after the participants chose the last (100^th^) card.

Data from the field test of FUT-SAT were recorded with a digital camera (SONY HDR-XR100). The digital videos were transferred to a laptop (COMPAQ 510 processor Intel Core 2 Duo) via cable and converted into avi. files. The software Soccer Analyzer was used for data processing. This software inserts spatial references in field test video and permits the identification of the positions and movements of players on the field. Data collection for the IGT was carried out using two laptops (COMPAQ 510 processor Intel Core 2 Duo and HP Pavilion dv4 14300us). These data were stored on a laptop and later analyzed.

The values for offensive, defensive and game tactical behaviors were recorded according to the accurate rate of tactical actions performed by players in the field test, which was provided by the output of the test. The participants were grouped according to low, intermediate and high levels of offensive, defensive and game tactical behavior, as defined by their accuracy rates. In the low group were the players who achieved scores ≤25%; in the high group were those with scores ≥75%. The intermediate group (>25% and <75%) was not considered in the analysis. Descriptive values for these groups are shown in Table 1. Data analysis of IGT was accomplished using results provided by the program used in data collection. The performance of participants was measured using the IGT net score provided by the test output.

**Table 1:** Descriptive values of the tactical behavior of participants.

Tactical Variables	Low group		High group		P
	Mean	SD	Mean	SD	
OTB1	73.92	7.89	96.97	2.25	p<.001
DTB2	59.69	9.83	92.32	3.71	p<.001
GTB3	69.70	6.66	92.83	1.99	p<.001

p<.05.

OTB: Offensive Tactical Behavior; DTB: Defensive Tactical Behavior; GTB: Game Tactical Behavior; SD: standard deviation.

### Data Analyses

Descriptive statistics were used to verify the means and standard deviations of offensive, defensive and game tactical behaviors. Values of quartiles were also obtained. The normality of the data distributions was verified by the Kolmogorov-Smirnov test. The low and high groups were compared and found to be different. Comparisons of the performance in IGT net score between the low and high groups for offensive, defensive and game tactical behaviors were accomplished using the non-parametric Mann-Whitney test. The effect size analysis for the Mann-Whitney was calculated using the following equation: 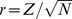 where, r is the effect size, Z is the z-score, and N is the overall number of cases.

The test-retest method was used to verify the coefficient of reliability of the tactical analysis [25]. A minimum of three weeks elapsed between analyses. Ten trained observers evaluated a total of 1,583 tactical actions (16.3%), a value higher than the minimum recommended (10%) by the literature [26]. Values of intra-observer reliability varied from 0.79 (SE = 0.053) to 1.00, and values of inter-observer reliability varied between 0.71 (SE = 0.013) and 0.85 (SE = 0.017). The statistic of Kappa was used to verify the coefficient of reliability of the analysis.

Statistical analyses were conducted using the Statistical Package for Social Sciences (SPSS) 18.0. The level of significance used was p<0.05.

## Results

**Figure 1:**
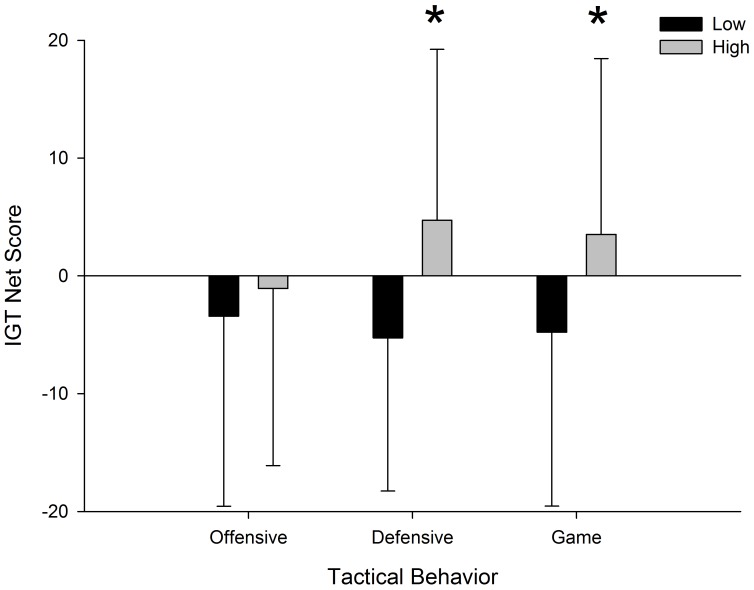
Comparison of the performance of players from low and high tactical behavior groups on IGT net score.

Figure 1 presents the performance of players on the IGT. Comparisons of the IGT net scores achieved by players from low (≤25%) and high (≥75%) Offensive, Defensive and Game Tactical Behavior are shown.

Differences between the low and high groups on the IGT net scores were observed with regard to Defensive Tactical Behavior (DTB) (low group (M = −5.263; SD = 12.998); high group (M = 4.718; SD = 14.513); (Z = −3.113; p = 0.002; r = −0.355) and Game Tactical Behavior (low group (M = −4.790; SD = 14.752); high group (M = 3.526; DP = 14.926); (Z = −2.267; p = 0.023; r = −0.260)).

## Discussion

The present study aimed to explore the influence of affective decision-making on tactical behavior in Under-15 soccer players. Statistically significant differences were observed in IGT net scores between players with low and high defensive tactical behavior (DTB) and game tactical behavior (GTB). These results revealed that affective decision-making ability was different between the lower and higher groups and may have influenced the tactical behavior of the players, specifically in the defensive phase and in the game itself. Thus, affective decision-making was shown to be an important measure in estimating the ability of young soccer players to achieve high levels of defensive and game tactical behavior.

No statistically significant differences in IGT net scores were observed between players in the low and high groups with regard to offensive tactical behavior (OTB). This result revealed that in the offensive phase, affective decision-making seems not to influence the tactical behavior of players. It is possible that in this phase of the game, differences in tactical behavior of the players is influenced by others factors, such as technical, perceptual and cognitive skills, among others. Differences in the characteristics of the two phases of soccer games are reported in the literature. In the defensive phase of the game, low decision-making is more related to low performance since that players prioritize order, organization and security [27]. In contrast, in offensive phase of the game, risk-taking behavior seems to be not so deleterious. In the defensive phase, players are pressured all the time to not make mistakes, whereas this pressure is less because an error in attack is not so harmful in the offensive phase. Thus, players can present lower levels of affective decision-making ability and even achieve great OTB, whereas this deficiency would more likely detract from DTB. In practice, in the offensive phase of play, the tactical behavior of players should not be influenced by their affective decision-making. Therefore, good or bad players with this ability could achieve high levels of OTB. However, this ability seems to be very important for players achieving high DTB. Thus, it is recommended that players with good performance on affective decision-making focus on defense because they are expected to achieve higher DTB than those players with poor affective decision-making performance.

Affective decision-making has been previously investigated in neuropsychological clinical settings, and it has been shown to be important for evaluating cognitive damage in patients [23], [28], [29]. A group of core neuropsychological abilities have been categorized as Executive Functions (EF). Some of these abilities, termed “cool” EF, are related to attention, working memory, planning and inhibition and are mediated by lateral inferior and dorsolateral frontostrial and frontoparietal networks [18]. Affective decision-making has been classified as “hot” EF, which is associated with events that have emotionally significant consequences and are mediated by the lateral orbitofrontal and ventromedial frontal regions of the prefrontal cortex [16].

A previous study by Vestberg et al. [21] revealed that some cognitive abilities, such as creativity, response inhibition and cognitive flexibility, can predict the level of performance of soccer players. All of these abilities are categorized as “cool” EF. The present study is the first to provide evidence on the influence of affective decision-making on tactical behavior of players. The results revealed that players with better affective decision-making were also better in DTB and GTB.

To achieve successful tactical behavior in soccer, players must perform suitable positioning and distribution on the game field, anticipate actions and make appropriate decisions [30], [31]. Such decisions are influenced by some individual and contextual factors that guide them to make the best tactical decisions according to game constraints [32] and are also influenced by some motivational and emotional features [17]. According to the somatic marker hypothesis, decisions made by individuals are influenced by marker signals that arise through bioregulatory processes, including those that are expressed through emotions and feelings, which often occur in situations where individuals are faced with situations resulting in gains and losses as well as risks and rewards, all of which are very common in soccer games [33].

A wrong pass, an error in positioning or any wrong tactical decision made by a player can result in a loss for the team. It is not difficult to remember situations in official matches when a wrong decision by a player, specifically in defense, had a negative consequence for his team. In contrast, an unpredictable and successful offensive decision can result in goals scored and winning. In some situations, impulsive decision-making can result in effective offensive tactical behaviors. It is possible that because of such situations, affective decision-making did not differ between low versus high players with regard to OTB.

During a soccer game, players are faced with situations in which they must choose, among several possibilities, the best and safest to achieve their goals with less or no risk(s). Through qualified training, players can explore the various possibilities of the game and learn to recognize and organize appropriate configurations of play [7]. To perform successfully, players must develop multiple abilities, including affective abilities. Although neuropsychological studies reveal that considerable biological maturation of the frontal lobes occurs during childhood and early adolescence, there is evidence that this process continues slowly throughout late adolescence [34], [35].

Adolescence is a phase of development through the lifespan in which an increase in the expression of impulsive behavior is often observed. According to Erst [36], the motivated behavior in adolescence could be explained by imbalance between three neurobiological systems. The first system is related to avoidance of negative events processed under activation of brain structures such as the amygdala, insula and hippocampus. The second system is related to approach and exploratory behavior, including decisions based on novelty, immediate gains and pleasure. This system is related to the ventral striatum and orbitofrontal cortex. Finally, a third system related to regulation and balance between the former systems is related to the activation of the dorsolateral, ventromedial and anterior cingulate prefrontal cortex. The immaturity of structures related to the regulatory system during the adolescence and the hyperactivation of the approach system in comparison to the avoidance system is an explanation for the natural increase of the impulsive/appetitive/immediatist behavior in adolescents [37]. This behavioral trend decreases until the end of adolescence and the early adulthood and is related to the maturation of the prefrontal cortex.

Several studies support this proposal. For example, Smith, Xiao and Bechara [29] showed that performance on IGT was impaired in early adolescence (12 years old) but improved during last adolescence (up to age 17). These data suggest that the ability of taking the future into account during affective decision-making processes develops throughout adolescence. This difference between decision-making processes between adolescents and adults hinders the generalization of the presented results. Future research should be addressed considering the relationship between decision-making and tactical performance in soccer players during adulthood. According to Steinberg [38], emotions have an important impact on basic cognition. Therefore, this relationship may explain why affective ability is important for players to perform successful tactical behaviors. In this sense the present study point to the importance of the development of affective decision-making process development in adolescent players.

This study presents important findings on the role of affective decision-making for tactical behaviors in soccer players. Such findings could help to highlight functions performed by the complex neurocognitive system and their role in supporting players’ abilities to achieve successful performance. This information could also be useful to technical committee professionals in the process of identification and development of young soccer players. Greater affective decision-making abilities were related to better tactical behavior in young soccer players; thus, affective decision-making must be developed in training.

It is important to affirm that the task used to assess affective decision-making in the present study is neither specific nor ecologically related to the game of soccer. However, the findings presented here are important because they reveal that the assessment of affective decision-making by a validated neuropsychological test can estimate players’ potential to perform efficient tactical behaviors. Additional research involving players from different age categories and levels of competitiveness could also increase the impact of the findings of this study.

A possible limitation of this study is that we did not control for the level of education/academic achievement and for years of practice. However, we used a sample with low standard deviation (0.63) with respect to age. In addition, the entire sample received formal education up to the date of data collection.

## Conclusions

From the results observed in this study, it is possible to affirm that tactical behavior influences affective decision-making in under-15 soccer players. It was found that differences in performance on the Iowa Gambling Task (IGT) neuropsychological test were linked to the tactical behavior scores of players. Players with high Defensive and Game Tactical Behavior presented better performance on IGT than those with low Defensive and Game Tactical Behavior. Such findings support the statement that affective decision-making is an important measure for predicting the level of tactical behavior to be achieved by young soccer players. Data from this study highlight the importance of developmental factors in soccer players, but there is a need for additional studies that analyze the influence of affective decision-making on the tactical behavior of young soccer players of different age categories and levels of competitiveness.

## References

[1] Lees A, Nolan L (1998) The biomechanics of soccer: a review. Journal of Sports Sciences 16: 211–234. 9596356 10.1080/026404198366740

[2] Ekblom B (1986) Applied physhiology of soccer. Sports Medicine 3: 50–60. 3633120 10.2165/00007256-198603010-00005

[3] Shephard RJ (1999) Biology and medicine of soccer: an update. Journal of Sports Sciences 17: 757–786. 10573331 10.1080/026404199365498

[4] Elferink-Gemser MT, Visscher C, Richart H, Lemmink K (2004) Development of the Tactical Skills Inventory for Sports 1. Perceptual and Motor Skills 99: 883–895. 15648483 10.2466/pms.99.3.883-895

[5] Gréhaigne JF, Godbout P (1995) Tactical knowledge in team sports from a constructivist and cognitivist perspective. Quest 47: 490–505.

[6] Grehaigne JF, Bouthier D, David B (1997) Dynamic-system analysis of opponent relationships in collective actions in soccer. Journal of Sports Sciences 15: 137–149. 9258844 10.1080/026404197367416

[7] Gréhaigne JF, Godbout P, Bouthier D (2001) The teaching and learning of decision making in team sports. Quest 53: 59–76.

[8] McPherson SL (1994) The development of sport expertise: Mapping the tactical domain. Quest 46: 223–240.

[9] Williams A, Davids K (1995) Declarative knowledge in sport: A by-product of experience or a characteristic of expertise? Journal of Sport and Exercise Psychology 17: 259–275.

[10] Kannekens R, Elferink-Gemser MT, Visscher C (2009) Tactical skills of world-class youth soccer teams. Journal of Sports Sciences 27: 807–812. 19437183 10.1080/02640410902894339

[11] Teoldo I, Garganta J, Greco PJ, Mesquita I, Seabra A (2010) Influence of Relative Age Effects and Quality of Tactical Behaviour in the Performance of Youth Soccer Players. International Journal of Performance Analysis in Sport 10: 82–97.

[12] Lage GM, Gallo LG, Cassiano GJ, Lobo IL, Vieira MV, et al (2011) Correlations between impulsivity and technical performance in handball female athletes. Psychology 2: 721–726.

[13] Casanova F, Oliveira J, Williams M, Garganta J (2009) Expertise and perceptual-cognitive performance in soccer: a review. Revista Portuguesa de Ciências do Desporto 9: 115–122.

[14] Chan RC, Shum D, Toulopoulou T, Chen EY (2008) Assessment of executive functions: Review of instruments and identification of critical issues. Archives of Clinical Neuropsychology 23: 201–216. 18096360 10.1016/j.acn.2007.08.010

[15] Stuss DT, Anderson V (2004) The frontal lobes and theory of mind: Developmental concepts from adult focal lesion research. Brain and Cognition 55: 69–83. 15134844 10.1016/S0278-2626(03)00271-9

[16] Kerr A, Zelazo PD (2004) Development of “hot” executive function: The children’s gambling task. Brain and cognition 55: 148–157. 15134849 10.1016/S0278-2626(03)00275-6

[17] Bechara A (2004) The role of emotion in decision-making: evidence from neurological patients with orbitofrontal damage. Brain and Cognition 55: 30–40. 15134841 10.1016/j.bandc.2003.04.001

[18] Zelazo PD, Müller U (2002) Executive function in typical and atypical development. In: Goswami U, editor. Blackwell Handbook of Childhood Cognitive Development. Oxford: Blackwell. 445–469.

[19] Garon N, Moore C (2004) Complex decision-making in early childhood. Brain and Cognition 55: 158–170. 15134850 10.1016/S0278-2626(03)00272-0

[20] Prencipe A, Kesek A, Cohen J, Lamm C, Lewis MD, et al (2011) Development of hot and cool executive function during the transition to adolescence. Journal of Experimental Child Psychology 108: 621–637. 21044790 10.1016/j.jecp.2010.09.008

[21] Vestberg T, Gustafson R, Maurex L, Ingvar M, Petrovic P (2012) Executive functions predict the success of top-soccer players. PloS one 7: e34731. 22496850 10.1371/journal.pone.0034731 PMC3319604

[22] Teoldo I, Garganta J, Greco PJ, Mesquita I, Maia J (2011) System of tactical assessment in Soccer (FUT-SAT): Development and preliminary validation. Motricidade 7: 69–83.

[23] Bechara A, Tranel D, Damasio H (2000) Characterization of the decision-making deficit of patients with ventromedial prefrontal cortex lesions. Brain 123: 2189–2202. 11050020 10.1093/brain/123.11.2189

[24] Malloy-Diniz LF, Leite WB, Moraes PHP, Correa H, Bechara A, et al (2008) Brazilian Portuguese version of the Iowa Gambling Task: transcultural adaptation and discriminant validity. Revista Brasileira de Psiquiatria 30: 144–148. 18470405 10.1590/s1516-44462008005000009

[25] Robinson G, O’Donoghue P (2007) A weighted kappa statistic for reliability testing in performance analysis of sport. International Journal of Perfomance Analysis in Sport 7: 12–19.

[26] Tabachnick B, Fidell L (2007) Using Multivariate Statistics. Nova Yorque: Harper and Row Publishers. 1008 p.

[27] Gréhaigne J-F, Godbout P, Zerai Z (2011) How the “rapport de forces” evolves in a soccer match: the dynamics of collective decisions in a complex system. Revista de Psicología del Deporte 20: 747–765.

[28] Torralva T, Kipps CM, Hodges JR, Clark L, Bekinschtein T, et al (2007) The relationship between affective decision-making and theory of mind in the frontal variant of fronto-temporal dementia. Neuropsychologia 45: 342–349. 16893555 10.1016/j.neuropsychologia.2006.05.031

[29] Smith DG, Xiao L, Bechara A (2012) Decision making in children and adolescents: Impaired iowa gambling task performance in early adolescence. Developmental Psychology 48: 1180. 22081879 10.1037/a0026342

[30] Roca A, Ford PR, McRobert AP, Williams AM (2011) Identifying the processes underpinning anticipation and decision-making in a dynamic time-constrained task. Cognitive Processing 12: 301–310. 21305386 10.1007/s10339-011-0392-1

[31] Sampaio J, Maçãs V (2012) Measuring tactical behaviour in football. International Journal of Sports Medicine 33: 395–401. 22377947 10.1055/s-0031-1301320

[32] Araújo D, Davids K, Hristovski R (2006) The ecological dynamics of decision making in sport. Psychology of Sport and Exercise 7: 653–676.

[33] Bechara A, Damasio H, Damasio AR (2000) Emotion, decision making and the orbitofrontal cortex. Cerebral Cortex 10: 295–307. 10731224 10.1093/cercor/10.3.295

[34] Stuss DT (1992) Biological and psychological development of executive functions. Brain and cognition 20: 8–23. 1389124 10.1016/0278-2626(92)90059-u

[35] Anderson VA, Anderson P, Northam E, Jacobs R, Catroppa C (2001) Development of executive functions through late childhood and adolescence in an Australian sample. Developmental Neuropsychology 20: 385–406. 11827095 10.1207/S15326942DN2001_5

[36] Ernst M (In Press) The triadic model perspective for the study of adolescent motivated behavior. Brain and Cognition. 10.1016/j.bandc.2014.01.006 PMC4248307 24556507

[37] Ernst M, Pine D, Hardin M (2006) Triadic model of the neurobiology of motivated behavior in adolescence. Psychological Medicine 36: 299–312. 16472412 10.1017/S0033291705005891 PMC2733162

[38] Steinberg L (2005) Cognitive and affective development in adolescence. Trends in Cognitive Sciences 9: 69–74. 15668099 10.1016/j.tics.2004.12.005

